# CXCL13, CXCL10 and CXCL8 as Potential Biomarkers for the Diagnosis of Neurosyphilis Patients

**DOI:** 10.1038/srep33569

**Published:** 2016-09-21

**Authors:** Cuini Wang, Kaiqi Wu, Qian Yu, Sufang Zhang, Zixiao Gao, Yudan Liu, Liyan Ni, Yuanyuan Cheng, Zhifang Guan, Mei Shi, Haikong Lu, Yongliang Lou, Pingyu Zhou

**Affiliations:** 1 STD Institute, Shanghai Skin Disease Hospital, Shanghai, P. R. China; 2 School of Laboratory Medicine, Wenzhou Medical University, Wenzhou, Zhejiang, China; 3 Shanghai Skin Disease Hospital, Clinical School of Anhui Medical University, Shanghai, P. R. China

## Abstract

At present, diagnosis for neurosyphilis remains a major clinical challenge. Venereal Disease Research Laboratory (VDRL) titer of the cerebrospinal fluid (CSF) is suboptimally sensitive to diagnose neurosyphilis, which can be negative in neurosyphilis patients, especially in asymptomatic neurosyphilis patients. In the search for biomarkers of neurosyphilis, we investigated the chemokine profile in CSF of neurosyphilis patients and found that the concentrations of CXCL13, CXCL10 and CXCL8 were selectively elevated in neurosyphilis patients and correlated with CSF protein concentration and CSF-VDRL titer. After antibiotic treatment, the concentration of these chemokines was dramatically reduced. The area under the ROC curve (AUC) of CSF CXCL13, CXCL8,CXCL10 and the CSF/serum ratio of CXCL13, CXCL8,CXCL10 in the diagnosis of neurosyphilis were 0.940, 0.899, 0.915, 0.963, 0.846 and 0.926, respectively. The corresponding sensitivities/specificities of CSF CXCL13, CXCL8,CXCL10 and the CSF/serum ratio of CXCL13, CXCL8,CXCL10 in diagnosis of neurosyphilis were 85.4%/89.1%, 79%/90.1% and 79.6%/91.1%, 86.6%/99%, 79%/73.3% and 86%/92.1%, respectively. Our results suggest that the elevated concentrations of CXCL13, CXCL8, and CXCL10 or their increasing CSF/serum ratios may be potential biomarkers of neurosyphilis, particularly for asymptomatic neurosyphilis. Reduced concentration of these chemokines may indicate the prognosis of antibiotic therapy.

Syphilis, a sexually transmitted multi-stage disease caused by *Treponema pallidum* subsp. *Pallidum*, remains to be a global public health problem. The World Health Organization estimates that 10–12 million new infections occur each year, primarily in low and middle-income countries^1^. In 2014, 419,091 new syphilis cases were reported in China^2^. *T. pallidum* can disseminate to the central nervous system (CNS) within days after exposure and can lead to asymptomatic meningitis that, if left untreated, can progress to severe and irreversible symptomatic neurosyphilis. Therefore, identification of sensitive biomarkers to facilitate neurosyphilis diagnosis would be valuable for preventing serious sequelae. Unfortunately, there is no “gold standard” for the diagnosis of neurosyphilis. The presence of a reactive Venereal Disease Research Laboratory (VDRL) in the cerebrospinal fluid (CSF) is specific, but the generally accepted sensitivity of CSF-VDRL is 30 to 70%^3^,^4^. A reactive VDRL may establish a diagnosis of neurosyphilis while a negative VDRL cannot exclude the possibility of neurosyphilis. Especially for the diagnosis of early asymptomatic neurosyphilis, CSF VDRL test is insensitive^3^,^4^. *T. pallidum* was isolated from cerebrospinal fluid by rabbit infection test of 12 (30%) of 40 patients with primary and secondary syphilis, but the CSF VDRL was positive in only four (33%) of these 12 patients^5^. Therefore, more accurate and sensitive biomarkers other than a reactive CSF-VDRL are needed.

Chemokines and their receptors are expressed in both neuronal and glial cells of the CNS. Elevated levels of various chemokines have been found in CSF and been associated with the severity or progression of several CNS inflammatory disorders^6^,^7^,^8^. CSF CXCL13 level was significantly increased and has been suggested as an early marker of Lyme neuroborreliosis^9^,^10^, indicating increased levels of chemokines as potential diagnostic biomarkers of certain infectious diseases. Previous studies have shown that the concentration of CSF CXCL13 was significantly elevated and had added value for the diagnosis of neurosyphilis in HIV co-infected syphilis patients, suggesting that chemokines may be involved in the inflammatory disorder of CNS in neurosyphilis patients^11^,^12^,^13^.

In this study, we analyzed the global expression profiles of 36 chemokines in CSF of neurosyphilis patients for the purpose of screening novel biomarkers as a diagnosis of neurosyphilis in HIV-negative syphilis patients, especially in asymptomatic neurosyphilis patients.

## Results

### The levels of CSF CXCL13, CXCL8 and CXCL10 were selectively elevated in neurosyphilis patients by chemokine antibody array

**Figure 1:**
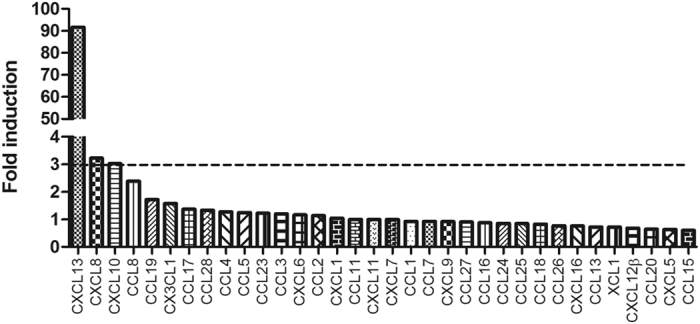
CSF CXCL13, CXCL10 and CXCL8 levels were selectively upregulated in neurosyphilis patients. Chemokine array was used to profile 36 chemokines in the CSF of 9 neurosyphilis and 7 non-neurosyphilis patients. The mean value of each chemokine was defined for the non-neurosyphilis patients, and fold induction was calculated for neurosyphilis patients. CXCL12αand CCL22 were undetectable.

To investigate the expression profiles of chemokines in CSF of neurosyphilis patients, the levels of 36 chemokines in the CSF of 9 neurosyphilis patients (5 symptomatic neurosyphilis, 4 asymptomatic neurosyphilis) and 7 non-neurosyphilis patients (1 primary syphilis, 3 secondary syphilis, 3 latent syphilis) were examined using the chemokine antibody array. As shown in Fig. 1, notably, compared to non-neurosyphilis patients, the average levels of CXCL13, CXCL8 and CXCL10 in neurosyphilis patients were 91.64 (ranged from 5.89 to 484.5), 3.22 (ranged from 1 to 12.8), 3.02 (ranged from 1.68 to 9.46) fold higher than those in non-neurosyphilis patients (p < 0.001), respectively. CSF CCL20, CCL15 and CXCL5 levels were down-regulated in neurosyphilis patients compared to those of non-neurosyphilis patients (with at least a 0.65-fold decrease, p < 0.05). CXCL12α and CCL22 could not be detected in all patients.

### The CSF CXCL13, CXCL10 and CXCL8 levels were significantly higher in neurosyphilis patients than those in non-neurosyphilis patients

**Figure 2:**
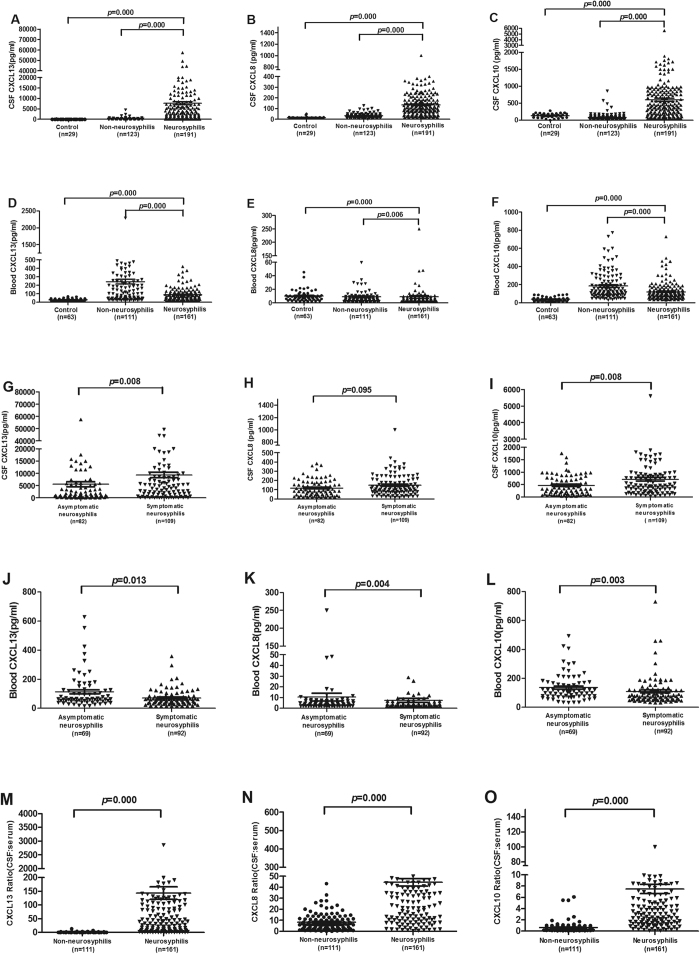
The levels of CSF CXCL13, CXCL10 and CXCL8 were significantly higher in neurosyphilis patients than those in neurosyphilis patients. (**A**) The level of CSF CXCL13 in control group (n = 29), non-neurosyphilis patients (including primary, secondary, latent and serofast syphilis; n = 123), neurosyphilis patients (including asymptomatic and symptomatic patients,n = 191). (**B**) The level of CSF CXCL8 among the samples shown in (**A**). (**C**) The level of CSF CXCL10 among the samples shown in (**A**). (**D**) The level of serum CXCL13 in control group (n = 63), non-neurosyphilis patients (including primary, secondary, latent and serofast syphilis; n = 111), neurosyphilis patients (including asymptomatic and symptomatic patients, n = 161). (**E**) The level of serum CXCL10 among the samples shown in (**D**). (**F**) The level of serum CXCL10 among the samples shown in (**D**). (**G**) The level of CSF CXCL13 in asymptomatic (n = 82) and symptomatic neurosyphilis patients (including meningovascular, paretic, tabetic and ocular, n = 109). (**H**) The level of CSF CXCL8 among the samples shown in (**G**). (**I**) The level of CSF CXCL10 among the samples shown in (**G**). (**J**) The level of serum CXCL13 in asymptomatic (n = 69), symptomatic neurosyphilis patients (including meningovascular, paretic, tabetic and ocular, n = 92). (**K**) The level of serum CXCL10 among the samples shown in (**J**). (**L**) The level of serum CXCL10 among the samples shown in (**J**). (**M**) The CSF: serum of CXCL13 in non-neurosyphilis patients (including primary, secondary, latent and serofast syphilis; n = 111), neurosyphilis patients (including asymptomatic and symptomatic patients, n = 161). (**N**) The CSF: serum of CXCL8 among the samples shown in (**M**). (**O**) The CSF: serum of CXCL10 among the samples shown in (**M**). Differences between groups were analyzed by using Mann-Whitney U test.

To ascertain the absolute levels of CXCL13, CXCL8 and CXCL10 in CSF and serum of syphilis patients, quantitative ELISA assays were performed to examine the levels of these chemokines in neurosyphilis (n = 191) and non-neurosyphilis patients (n = 123). Consistent with the antibody array data, the levels of CSF CXCL13, CXCL8 and CXCL10 were 45.9 (ranged from 0.09 to 341.42), 4.3 (ranged from 0.43 to 31.96), 7.2 (ranged from 0.37 to 67.16) fold higher in neurosyphilis patients than those in non-neurosyphilis patients, respectively. The analysis demonstrated that CSF CXCL13, CXCL8 and CXCL10 concentrations did not vary significantly even after adjusting for age and sex. The CSF CXCL13 level in neurosyphilis patients (median 1905.7 pg/ml, ranged from 15.6 to 57526.1 pg/ml) was the most significantly elevated chemokine in comparison with that in non-neurosyphilis patients (median 15.6 pg/ml, ranged from 15.6 to 4564.39 pg/ml, *p* = 0.000) (Fig. 2A). We then further compared the level of CSF CXCL13 between asymptomatic and symptomatic neurosyphilis patients and found that CSF CXCL13 level in symptomatic neurosyphilis was significantly higher than that in asymptomatic neurosyphilis patients (*p* = 0.008) (Fig. 2G). However, the serum CXCL13 level was significantly higher in non-neurosyphilis patients than that in neurosyphilis patients (*p* = 0.000) (Fig. 2D). Of note, the serum CXCL13 level was lower in symptomatic neurosyphilis patients than that in asymptomatic neurosyphilis patients (*p* = 0.013) (Fig. 2J). Thus, the CSF/serum ratio of CXCL13 in neurosyphilis patients (median 48.35, ranged from 0.09 to 2854.3) was greatly higher than that in non-neurosyphilis patients (median 0.32, ranged from 0.02 to 13.6, *p* = 0.000) (Fig. 2M).

Markedly elevated CSF concentrations of CXCL8 were detected in neurosyphilis patients (median 104.55 pg/ml, ranged from 13.59 to 1000 pg/ml) in comparison to that in non-neurosyphilis patients (median 26.28 pg/ml, ranged from 2 to 127.47 pg/ml, *p* = 0.000) (Fig. 2B), but the difference was not statistically different (*p* = 0.095) between symptomatic and asymptomatic neurosyphilis patients (Fig. 2H). The serum CXCL8 level was higher in non-neurosyphilis patients than that in neurosyphilis patients (*p* = 0.006) (Fig. 2E), and was also higher in asymptomatic neurosyphilis patients than that in symptomatic neurosyphilis patients (*p* = 0.004) (Fig. 2K). The CSF/serum ratio of CXCL8 (median 33.59, ranged from 0.51 to 220.1) in neurosyphilis patients was significantly higher than that in non-neurosyphilis patients (median 6.1, ranged from 0.02 to 43.3, *p* = 0.000) (Fig. 2N).

The CSF CXCL10 level in neurosyphilis patients (median 426.55 pg/ml, ranged from 31.2 to 5603.2 pg/ml) were significantly higher than that in non-neurosyphilis patients (median 44.76 pg/ml, ranged from 31.2 to 859.2 pg/ml, *p* = 0.000) (Fig. 2C). Signifincalty higher level as also found in patients with symptomatic neurosyphilis than otherwise (*p* = 0.008) (Fig. 2I). On the contrary, the serum CXCL10 level was higher in non-neurosyphilis patients than that in neurosyphilis patients (Fig. 2F), and was also higher in asymptomatic neurosyphilis patients than that in symptomatic neurosyphilis patients (*p* = 0.003) (Fig. 2L). The CSF/serum ratio of CXCL10 in neurosyphilis patients (median 4.13, ranged from 0.07 to 99.9) was significantly higher than that in non-neurosyphilis patients (median 0.4, ranged from 0.06 to 6.08, *p* = 0.000) (Fig. 2O).

### CSF CXCL13, CXCL10 and CXCL8 levels positively correlate with CSF protein concentration and CSF-VDRL titer in neurosyphilis patients

**Figure 3:**
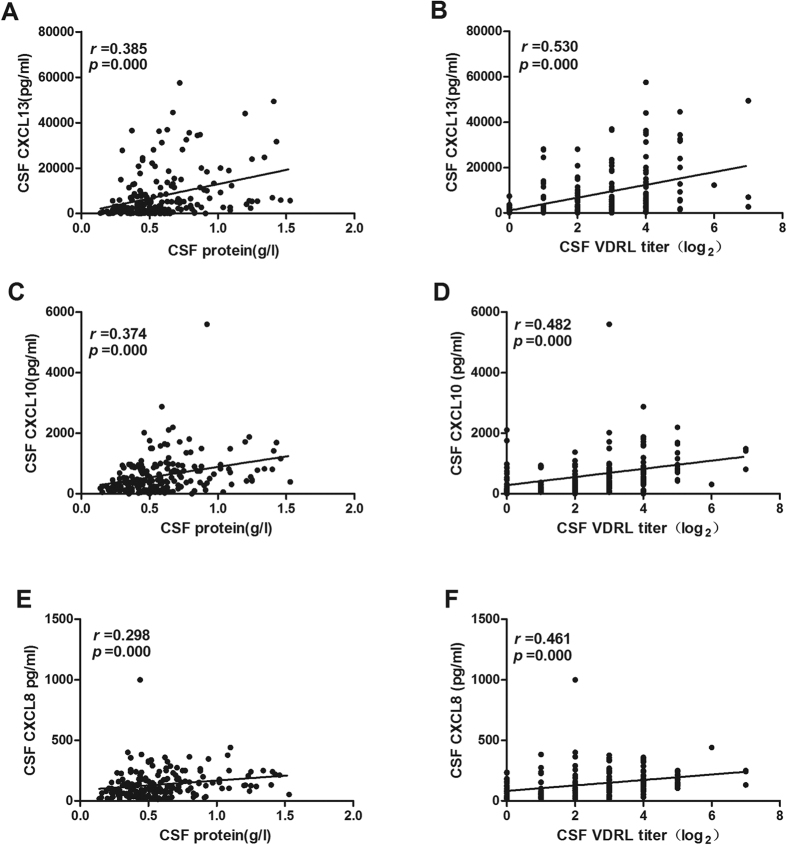
Correlations between the CSF CXCL13, CXCL10, CXCL8 levels and CSF protein concentration, CSF VDRL titer in neurosyphilis patients. CSF protein concentration (**A**), CSF VDRL titer (**B**) are plotted against CSF CXCL13 level in neurosyphilis patients (n = 191); CSF protein concentration (**C**), CSF VDRL titer (**D**) are plotted against CSF CXCL10 level in neurosyphilis patients (n = 191); CSF protein concentration (**E**), CSF VDRL titer (**F**) are plotted against CSF CXCL8 level in neurosyphilis patients (n = 191). Each dot represents an individual patient. The straight line in each graph is the result of linear regression analysis. Spearman correlation analysis was performed between the level of CSF CXCL13, CXCL8, CXCL10 and other parameters. Spearman’s correlating coefficients (*r*) and *p* values are shown.

*T. pallidum* is capable of invading and damaging the central nervous system. CSF is abnormal in neurosyphilis patients, including a reactive CSF-VDRL, pleocytosis, and/or elevated protein concentration. Given the measurements were correlated with the disease activity, we therefore investigated the correlation between CSF CXCL13, CXCL10 and CXCL8 levels and these measurements in neurosyphilis patients. We found that CSF CXCL13 level was positively correlated with CSF protein level (*r* = 0.385, *p* = 0.000) (Fig. 3A) and CSF VDRL titer (*r* = 0.530, *p* = 0.000) (Fig. 3B). The CSF CXCL10 and CXCL8 levels were also correlated with the CSF protein levels (*r* = 0.374, *p* = 0.000; *r* = 0.298, *p* = 0.000, respectively) (Fig. 3C,E) and CSF VDRL titer(*r* = 0.482, *p* = 0.000; *r* = 0.461, *p* = 0.000, respectively) (Fig. 3D,F). There was no correlation between CSF CXCL13, CXCL10, CXCL8 levels and CSF WBC counts in neurosyphilis patients (data not shown).

### Decline of CSF CXCL13, CXCL10 and CXCL8 levels could serve as markers as effective of antibiotic treatment for neurosyphilis patients

**Figure 4:**
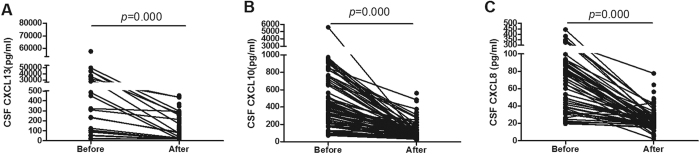
CSF CXCL13, CXCL10 and CXCL8 concentrations before and after treatment in neurosyphilis patients. (**A**) CSF CXCL13 levels before and after antibiotic treatment in neurosyphilis patients (n = 89). (**B**) CSF CXCL10 levels before and after antibiotic treatment in neurosyphilis patients (n = 89). (**C**) CSF CXCL8 levels before and after antibiotic treatment in neurosyphilis patients (n = 89). Differences between before and after treatment were analyzed by using paired *t*-test.

In 89 neurosyphilis patients who had one or more follow-up visits after treatment, CSF samples were collected after the first antibiotic treatment. As shown in Fig. 4, there was a sharp decline in CSF CXCL13 level after the first treatment (before treatment, median 3321 pg/ml, ranged from 15.6 to 57526.1 pg/ml; after treatment, median 53.6 pg/ml, ranged from 15.6 to 2000 pg/ml) (*p* = 0.000). The CSF CXCL10 and CXCL8 levels were also significantly decreased. The CSF CXCL10 and CXCL8 levels before treatment were 450.3 pg/ml (ranged from 72.1 to 5603.2 pg/ml) and 100.1 pg/ml (ranged from19.5 to 442.4 pg/ml), respectively, which were reduced to 91.5 pg/ml (ranged from 31.2 to 558.7 pg/ml)) (*p* = 0.000) and 23.6 pg/ml (ranged from 2 to 77.6 pg/ml) (*p* = 0.000) after treatment, respectively. These data indicated that CSF CXCL13, CXCL10 and CXCL8 levels may serve as markers for effective antibiotic treatment for neurosyphilis patients.

### Diagnostic values of CXCL13, CXCL10 and CXCL8 for neurosyphilis

Given the marked elevation of CSF CXCL13, CXCL10 and CXCL8 as well as CSF/serum ratio of these chemokines in neurosyphilis patients, we further evaluated these chemokines as biomarkers in neurosyphilis diagnosis using the receiver operating characteristic (ROC) curve analysis. The optimal cut-off values were defined by the sum of maximum sensitivity and specificity, which were 256.4 pg/ml, 163.1 pg/ml, 48.1 pg/ml for CSF titer and 4.36, 1.02 and 10.3 for the CSF/serum ratio of CXCL13, CXCL10, CXCL8, respectively. As shown in Table 1, CSF titers had 85.4%, 79.6%, and 79% sensitivity, and 89.1%, 91.1% and 90.1% specificity. The areas under the ROC curve (AUC) were 0.940, 0.915 and 0.899 (95% confidence interval [95% CI] 0.913–0.967, 0.881–0.950 and 0.863–0.935), respectively. The ratio of CSF/serum for CXCL10, CXCL8 and CXCL13 had 86%, 79% and 86.6% sensitivity, 92.1%, 73.3% and 99% specificity, and the AUCs of 0.926, 0.846 and 0.963 (95% CI 0.893–0.959, 0.799–0.892 and 0.942–0.983), respectively. CSF VDRL had 90.4% sensitivity, 100% specificity, and an AUC of 0.952 (95% CI 0.925–0.980). The AUCs of the CSF CXCL13, CXCL10 and the CSF/serum ratios of CXCL13, CXCL10 were compared with that of VDRL, and we found that they were not statistically different (*p* = 0.055, *p* = 0.050, *p* = 0.270, *p* = 0.108, respectively). The AUCs of CSF CXCL8 and the CSF/serum ratio of CXCL8 were lower than that of CSF VDRL (*p* = 0.002, *p* = 0.001, respectively).

CSF-VDRL titer is regarded as insensitive for the diagnosis of asymptomatic neurosyphilis^3^,^4^. We also estimated the diagnostic values of these biomarkers in asymptomatic neurosyphilis. As shown in Table 2, the AUCs were 0.922 (95% CI 0.880–0.965), 0.886 (95% CI 0.834–0.938), 0.866 (95% CI 0.808–0.923) for CSF CXCL13, CXCL10 and CXCL8 and were 0.941 (95% CI 0.901–0.981), 0.892 (95% CI 0.836–0.947), 0.803 (95% CI 0.730–0.875) for the CSF/serum ratio of CXCL13, CXCL10, CXCL8, respectively. The AUC of CSF VDRL was 0.890 (95% CI 0.829–0.950). The AUC of the CSF/serum ratio of CXCL13 was the greatest among these biomarkers, which was higher than that of CSF VDRL (*p* = 0.0232). The AUCs of the ratio of CSF/serum CXCL8 were lower than that of VDRL (*p* = 0.037). There was not statistically different between the other biomarkers and that of CSF VDRL (*p* > 0.05 for all). The specificities of the above biomarkers were close to or above 90%, except for CSF/serum ratio of CXCL8 (72.1%), as defined by these thresholds.

### Diagnostic models that combine CSF CXCL13, CXCL10 and CXCL8 and the ratios of CSF/serum CXCL13, CXCL10 and CXCL8

The combination assay of CSF CXCL13, CXCL10 and CXCL8 (Model 1) had 90.4% sensitivity, 82.9% specificity, and yielded an AUC of 0.949 (95% CI 0.926–0.972) for the diagnosis of neurosyphilis (Table 1), and 77.9% sensitivity, 92.6% specificity, and yielded an AUC of 0.925 (95% CI 0.885–0.964) for the diagnosis of asymptomatic neurosyphilis (Table 2). The AUC of Model 1 was higher than that of CSF CXCL10, CXCL8 for all neurosyphilis (*p* = 0.0165, *p* < 0.0001, respectively) and for asymptomatic neurosyphilis (*p* = 0.0363, *p* = 0.0047, respectively). But it was not statistically different compared with that of CSF CXCL13 and CSF VDRL for all neurosyphilis (*p* = 0.1167, *p* = 0.3237, respectively) and for asymptomatic neurosyphilis (*p* = 0.6399, *p* = 0.8994, respectively).

The combination assay of the ratios of CSF/serum CXCL13, CXCL10 and CXCL8 (Model 2) had 90.4% and 86.8% sensitivity, 93.7% and 94.6% specificity, and yielded the AUCs of 0.966 and 0.938 (95% CI 0.945–0.986 and 0.896–0.981) for the diagnosis of all neurosyphilis and asymptomatic neurosyphilis, respectively (Tables 1 and 2). The AUC of Model 2 was not statistically different from that of the ratio of CSF/serum CXCL13 and CSF VDRL (*p* = 0.3968, *p* = 0.1386, respectively), but it was higher than that of the ratio of CSF/serum CXCL8 and CXCL10 (*p* < 0.0001, *p* = 0.007, respectively) for all neurosyphilis. For asymptomatic neurosyphilis, the AUC of Model 2 was not statistically different from that of the ratio of CSF/serum CXCL13 (*p* = 0.6634), but it was higher than that of CSF VDRL (*p* = 0.0412). Importantly, the sensitivity of Model 2 reached to 86.8%, which was higher than that of CSF VDRL (*p* < 0.05) for the diagnosis of asymptomatic neurosyphilis.

## Discussion

Prompt diagnosis and treatment of neurosyphilis is important, because delayed treatment can lead to irreversible damage of CNS and inferior outcomes. Currently, the diagnosis of neurosyphilis is not difficult when patients have typical symptoms and signs. However, the diagnosis of asymptomatic neurosyphilis, especially presumptive neurosyphilis, which is mainly based on CSF abnormalities, is uncertain and difficult. Our results indicated that CXCL13 had good diagnostic accuracy for diagnosis of neurosyphilis, especially for asymptomatic neurosyphilis, which further strengthens the previous findings that CXCL13 is an excellent indicator for the diagnosis of neurosyphilis^11^,^12^,^13^.

CXCL13 is secreted by several cell types, including B cells, monocytes, dendritic cells, nerve cells, and microglial cells^14^,^15^,^16^. Spirochetal lipoproteins are critical for induction of CXCL13^17^, and the CXCL13 level can be increased in the early phases of infections^10^. In our study, elevated CSF and serum CXCL13 levels were observed in some secondary syphilis patients, but CSF TPPA was nonreactive, indicating that CXCL13 may be produced prior to the specific antibodies. Recent studies showed that the CSF CXCL13 level was elevated in HIV-positive neurosyphilis patients^11^,^12^,^13^, but in our study, the CSF CXCL13 were higher than that in HIV-positive neurosyphilis patients, indicating that CXCL13 may be affected by the immune status of HIV-infected patients.

CXCL10 and CXCL8 are inflammatory chemokines. CXCL10 is induced by IFN-γ, which can enhance innate antimicrobial defense, chemoattracts mononuclear cells and promotes adhesion of T cells^8^,^18^,^19^. CXCL8 can enhance microglial matrix metalloproteases production, leading to breakdown of the blood-brain barrier and invasion of inflammatory cells, thereby enhancing the inflammatory cascade that contributes to nerve damage^20^. The levels of CSF CXCL8 and CXCL10 have been shown to be significantly elevated in neuroborreliosis patients^21^,^22^. In our study, the levels of CSF CXCL8 and CXCL10 were increased in neurosyphilis patients, especially in symptomatic neurosyphilis patients. The levels of CSF CXCL10 and CXCL8 positively correlated with the CSF-VDRL titer and CSF protein concentration in neurosyphilis patients, indicating that CXCL8 and CXCL10 may be involved in the inflammatory process in neurosyphilis patients.

As the diagnostic biomarkers, the AUCs of CSF CXCL10 and CXCL8 levels and the CSF/serum CXCL10 ratio were more than or approximate to 0.9 for all untreated neurosyphilis patients, though they were lower than that of CSF VDRL. Combinatorial biomarkers can increase sensitivity or specificity^7^,^23^. In our study, CXCL13, CXCL10 and CXCL8 correlated significantly with each other. Therefore, multivariate logistic regression analysis was applied to build models based on these biomarkers for neurosyphilis diagnosis. Thus, we assessed the efficacy of combined CSF CXCL13, CXCL10 and CXCL8 (Model 1) and combined the ratio of CSF/serum CXCL13, CXCL10 and CXCL8 (Model 2) as biomarkers for diagnosis of neurosyphilis. For the diagnosis of all neurosyphilis, the AUCs and sensitivities of Model 1 and Model 2 were approximately equal to that of CSF VDRL. For asymptomatic neurosyphilis, the sensitivity of Model 2 reached 86.8% that was significantly higher than that of CSF VDRL. These results suggested that the diagnosis based on the combination of these biomarkers could improve the accuracy of diagnosis.

In addition, CSF VDRL may persist for years after successful treatment, whereas the levels of CSF CXCL13, CXCL10 and CXCL8 decrease rapidly after antibiotic treatment. Thus, these chemokines may be suitable for monitoring the treatment response, and differentiating an active infection from a past infection.

There were some limitations in our study. First, although this was the largest and most comprehensive analysis of candidate CSF biomarkers for neurosyphilis in HIV-negative patients to date, these biomarkers are also elevated in other inflammatory diseases, such as infectious diseases and some autoimmune disorders. However, these diseases were dramatically influenced by geography, history, serological testing, and MRI findings etc., which could easily be clinically differentiated from neurosyphilis. Second, since the levels of these biomarkers could decrease dramatically after the antibiotic treatment, one must carefully analyze the results according to patients’ medication history, especially patients with probable asymptomatic neurosyphilis. Third, in patients co-infected with HIV and syphilis, the presence of CXCL13, CXCL10 and CXCL8 in CSF could be associated with syphilis, HIV or both. We were unable to determine whether the optimal cut-off values were applicable to neurosyphilis in HIV-infected patients, and the efficacy of these three biomarkers still needs to be further determined.

In conclusion, our data show that CXCL13, CXCL10 and CXCL8 are potential biomarkers to be used as a complementary diagnostic tool for neurosyphilis. Similarly, they may be useful for monitoring therapeutic effect for neurosyphilis.

## Methods

### Ethics statement and Subjects

This study was performed at the Shanghai Skin Disease Hospital between Dec. 2010 and Dec. 2014, which was approved by the Ethics Committee of the Shanghai Skin Disease Hospital. All the methods were carried out in accordance with the approved guidelines. Written Informed consent was obtained from all participants. Patients were identified and referred for enrollment by the dermatologists, neurologists, psychiatrists and ophthalmologists after careful examinations and evaluations. The diagnosis of clinical stage of syphilis was determined based on a combination of a compatible history, clinical manifestations and the results of nontreponemal and treponemal tests in the serum and CSF. The diagnosis of syphilis included a reactive serum nontreponemal and treponemal test. Exclusion criteria included age  < 18 years, traumatic CSF collection, therapeutic intervention (except serofast) and co-infected with HIV.

In this study, two groups of patients were included: 1) neurosyphilis group (including 82 subjects with asymptomatic neurosyphilis, 4 subjects with meningovasculitis, 87 subjects with general paresis, 11 subjects with tabes dorsalis, and 7 subjects with ocular neurosyphilis); 2) non-neurosyphilis group with normal CSF WBC count, CSF protein concentration and CSF-VDRL negative (including 14 subjects with primary syphilis, 51 subjects with secondary syphilis, 42 subjects with latent syphilis, and 14 subjects with serofast syphilis, 2 ocular syphilis who had ocular signs or symptom but with normal CSF index). In addition, for measurement of the baseline level of the chemokines in blood, 63 healthy donors, who visited Shanghai Skin Disease Hospital voluntarily for a medical check-up for the purpose of STD prevention, were recruited to the study. Fresh blood was collected and serum was isolated from these volunteers. Since it is difficult to collect CSF from healthy donors, we used a separate control group of 29 patients who underwent orthopaedic or stone surgery (gall stone, kidney stone) but were serum RPR and TPPA negative, whose CSF samples were collected prior to spinal anaesthesia. The baseline levels of the chemokines in CSF were determined using samples from the control group. Further characteristics of the participants were presented in Table 3.

### Diagnostic criteria for neurosyphilis

The diagnosis of confirmed neurosyphilis included a reactive CSF-VDRL and a reactive CSF-TPPA in the absence of substantial contamination of CSF with blood. Presumptive neurosyphilis was defined as a nonreactive CSF-VDRL but a reactive CSF-TPPA with either or both of the following: (i) CSF protein concentration > 45 mg/dl and/or CSF white blood cell (WBC) count≥8/μl in the absence of other known causes for the abnormalities; (ii) clinical neurological or psychiatric manifestations consistent with neurosyphilis without other known causes for such abnormalities^24^,^25^.

Neurosyphilis is categorized as: asymptomatic, meningovascular, paretic, ocular and tabetic neurosyphilis. Asymptomatic neurosyphilis is defined by the presence of CSF abnormalities consistent with neurosyphilis and the absence of neurological/psychiatric symptoms or signs. Meningovasculitisis defined by clinical features of meningitis and magnetic resonance image (MRI) evidence of brain lesions and/or a stroke syndrome. General paresis is characterized by personality changes, dementia and psychiatric symptoms including mania or psychosis. Patients with sensory loss, ataxia, lancinating pains, bowel and bladder dysfunction were considered as tabes dorsalis. Ocular neurosyphilis is defined by the presence of CSF abnormalities consistent with neurosyphilis and ocular signs or symptoms (worsening visual acuity and visual fields, floaters, papillitis, uveitis). Patients with all these forms of neurosyphilis have no other known causes for these clinical abnormalities.

### The treatment and follow-up

According to Chinese National STI treatment Guidelines, neurosyphilis patients were given aqueous crystalline pencillin G, 4MU intravenously every 4 h for 14 days, or ceftriaxone intravenously with 2 g daily for 10 days if allergic to penicillin. Patients returned for follow-up visits at 3, 6, 9 and 12 months after treatment. All patients underwent lumbar puncture at the 3-month visit, and lumbar punctures were repeated at 6, 9 and 12 months if the previous follow-up CSF profiles were abnormal. Blood and CSF samples were collected at each follow-up visits.

### Chemokine Array Analysis

CSF samples were examined using the RayBiotech Human Chemokine Antibody Array G-Series 1 (RayBiotech, Norcross, GA), which can assess 36 chemokines in a batch including CXCL13, CCL28, CCL23, CCL27, CXCL16, CXCL5, CCL11, CCL24, CCL26, CX3CL1, CXCL6, CXCL1, CCL16, CCL1, CXCL8, CXCL10, CXCL11, XCL1, CCL2, CCL8, CCL7, CCL13, CCL22, CXCL9, CCL3, CCL4, CCL15, CCL20, CCL19, CXCL7, CCL18, CCL5, CXCL12α, CXCL12β, CCL17, CCL25. The assay was preformed according to the manufacturer’s instructions. The glass arrays were first blocked with blocking buffer and then incubated with CSF samples for 2 hours. They were then washed and incubated with biotinylated detection antibodies for another 2 hours, and subsequently with streptavidin-fluor for 2 hour. The density of image was quantified by a densitometer (LuxScan-10K/A, Capitalbio), and intensity was normalized with positive controls from the same glass array.

### Measurement of CXCL13, CXCL10 and CXCL8 in CSF and serum

CXCL13, CXCL10 and CXCL8 in CSF and serum were quantified by ELISA Kits according to manufacturer’s instructions (CXCL13 and CXCL10 ELISA kits were supplied by R&D Systems, CXCL8 ELISA kits were supplied by eBioscience). CXCL13, CXCL10 and CXCL8 concentration at or below the level of detection were set at the level of detection.

### Statistical analysis

Data are presented as median and interquartile range (IQR). Differences between groups were analyzed by using Mann-Whitney U test. Spearman correlation analysis was performed between the level of CSF CXCL13, CXCL8, CXCL10 and other parameters. Differences between before and after treatment were analyzed by using paired *t* test.

The ability of three biomarkers to diagnosis of neurosyphilis was evaluated by receiver-operating characteristic (ROC) curves. Sensitivities, specificities, positive predictive values (PPVs), negative predictive values (NPVs) were calculated using standard formulae and expressed as percent (95% CI).

We investigated the diagnostic performance of CSF CXCL13, CXCL8, CXCL10 and the ratio of CSF/serum CXCL13, CXCL10, CXCL8 for neurosyphilis diagnosis. Multivariate logistic regression analysis was applied and a model based on CSF CXCL13,CXCL10 and CXCL8 was built for neurosyphilis diagnosis, which was defined as Model1. The prediction rule was used to calculate a probability score for each patient based on their individual expression levels of the three biomarkers, where probability score = (0.001 × expression value of CSF CXCL13) + (0.004 × expression value of CSF CXCL10) + (0.013 × expression value of CSF CXCL8). The cut-off probability score in the combination detection assay was 1.1. According to the method as described above, we constructed a model combining the ratio of CSF/serum CXCL13, CXCL10 and CXCL8, defined as Model2, to derive a prediction rule in neurosyphilis. The probability score = (0.452 × expression value of CSF/serum CXCL13) + (0.425 × expression value of CSF/serum CXCL10) + (0.019 × expression value of CSF/serum CXCL8). The cut-off probability score in the combination detection assay was 2.23.

Statistical analyses were performed using SPSS 17.0 (SPSS Inc.,Chicago,IL,USA) and using MedCale version 8.0 (MedCale Software) for ROC curve analysis. Statistical significance in this study was set at *p* < 0.05 and all reported *p*-values were 2-sided.

## Additional Information

**How to cite this article**: Wang, C. *et al*. CXCL13, CXCL10 and CXCL8 as Potential Biomarkers for the Diagnosis of Neurosyphilis Patients. *Sci. Rep.* **6**, 33569; doi: 10.1038/srep33569 (2016).

## Figures and Tables

**Table 1:** Clinical diagnostic value of CSF chemokine biomarkers for neurosyphilis.

Biomarker	AUC*	95% CI#	Optimal cut-off	Sensitivity	Specificity
CSF CXCL13	0.940	0.913–0.967	256.4 pg/ml	85.4%	89.1%
CSF CXCL8	0.899	0.863–0.935	48.1 pg/ml	79%	90.1%
CSF CXCL10	0.915	0.881–0.950	163.1 pg/ml	79.6%	91.1%
CSF/Serum ratio of CXCL13	0.963	0.942–0.983	4.36	86.6%	99%
CSF/Serum ratio of CXCL8	0.846	0.799–0.892	10.3	79%	73.3%
CSF/Serum ratio of CXCL10	0.926	0.893–0.959	1.02	86%	92.1%
Model1	0.949	0.926–0.972	1.1	90.4%	82.9%
Model2	0.966	0.945–0.986	2.23	90.4%	93.7%
CSF VDRL	0.952	0.925–0.980	1	90.4%	100%

These biomarkers to diagnosis of neurosyphilis was evaluated by receiver-operating characteristic (ROC) curves.

AUC is the percentage of randomly drawn pairs for which the test is correct.

^#^CI, confidence interval.

**Table 2:** Sensitivities, specificities, positive predictive values and negative predict values of the biomarkers for asymptomatic neurosyphilis diagnosis.

Biomarker	AUC(95% CI)	Sensitivity(95% CI)	Specificity(95% CI)	PPV* (95% CI)	NPV^#^(95% CI)
CSF CXCL13	0.922 (0.880–0.965)	81.9 (71.6–89.2)	88.2 (80.5–93.4)	84.0 (73.8–90.8)	86.7 (78.7–92.1)
CSF CXCL8	0.866 (0.808–0.923)	71.1 (60.0–80.3)	89.1 (81.4–94.0)	83.1 (71.9–90.6)	80.3 (71.9–86.7)
CSF CXCL10	0.886 (0.834–0.938)	69.5 (58.2–78.9)	90.1 (82.6–94.7)	83.8 (72.4–91.3)	80.0 (71.7–86.4)
CSF/Serum CXCL13	0.941 (0.901–0.981)	82.9 (71.6–90.5)	99.0 (94.0–100)	98.3 (89.7–100)	89.6 (82.1–94.3)
CSF/Serum CXCL8	0.803 (0.730–0.875)	68.5 (56.4–78.6)	72.1 (62.3–80.2)	63.3 (51.6–73.6)	76.5 (66.7–84.3)
CSF/Serum CXCL10	0.892 (0.836–0.947)	77.0 (65.5–85.7)	92.7 (85.6–96.5)	87.7 (76.6–94.2)	85.6 (77.7–91.1)
Model1	0.925 (0.885–0.964)	77.9 (63.8–83.3)	92.6 (86.0–96.3)	87.3 (76.8–93.7)	84.2 (76.6–89.7)
Model2	0.938 (0.896–0.981)	86.8 (75.9–93.4)	94.6 (88.2–97.8)	90.8 (80.3–96.2)	92.2 (85.3–96.1)
CSF VDRL	0.890 (0.829–0.950)	77.9 (71.6–89.2)	100 (96.2–100)	100 (93.3–100)	89.1 (82.3–93.5)

*PPV, positive predictive value; ^#^NPV, negative predictive value; CI, confidence interval.

**Table 3:** Clinical and Laboratory Characteristics of Control and Syphilis Patients.

	Control 1 (blood) (n = 63)	Control 2 (CSF) (n = 29)	Non-neurosyphilis (n = 123)	Neurosyphilis	
				Asymptomatic (n = 82)	Symptomatic (n = 109)
Male (%)	48 (68.5)	18 (62.1)	71 (57.7)	52 (63.4)	98 (89.9)
Age (Median, IQR)	44 (25–55)	46 (27–61)	52.5 (42.25–59.5)	52 (39–59)	53.5 (47–59)
1/Serum RPR titer (median, IQR)	0 (0–0)	0 (0–0)	32 (8–64)	64 (32–128)	63 (32–64)
CSF-WBC, cells/μl (median, IQR)	ND	1 (0–5)	1.1 (0–4.3)	8.8 (2.2–39.05)	9.9 (2–35.8)
CSF protein, g/l (Median, IQR)	ND	0.23 (0.1–0.4)	0.29 (0.23–0.34)	0.43 (0.29–0.57)	0.63 (0.46–0.89)
CSF VDRL (+) (%)	ND	0 (0)	0 (0)	67 (81.7)	109 (100)
CSF VDRL (−) and CSF-TPPA (+) (%)	ND	0 (0)	35 (28.2)	15 (18.3)	0 (0)

Data are given as median (IQR), or frequencies.

Abbreviations: RPR, Rapid Plasma Regain test; CSF, cerebrospinal fluid; VDRL, Venereal Research Laboratory; TPPA, Treponema pallidum particle agglutination assay; WBC, white blood cells; ND, not done.
